# Hepatic Resection in Patients with Colo-Rectal Liver Metastases: Surgical Outcomes and Prognostic Factors of Single-Center Experience

**DOI:** 10.3390/jcm12062170

**Published:** 2023-03-10

**Authors:** Matteo Pagani, Rosita De Vincenti, Carolina Cecchi, Alice Apollinari, Benedetta Pesi, Francesca Leo, Sandro Giannessi, Massimo Fedi

**Affiliations:** Division of General Surgery, San Jacopo Hospital, 51100 Pistoia, Italy

**Keywords:** colorectal cancer, liver metastases, survival, liver resection, prognostic factors

## Abstract

Introduction: Surgical resection has a fundamental role in increasing the chance of survival in patients with colorectal liver metastases. The guidelines have been modified and expanded in time in order to increase the number of patients that can benefit from this treatment. The aim of this study is to analyze the main prognostic factors related to overall and disease-free survival of a series of consecutive patients undergoing liver resection for colorectal liver metastases (CRLM). Materials and Methods: A retrospective review of patients undergoing liver resection for CRLM between April 2018 and September 2021 was performed. Clinical data and laboratory parameters were evaluated using the log-rank test. OS and DFS were estimated using the Kaplan-Meier method. Results: A retrospective study on 75 patients who underwent liver resection for CRLM was performed. The OS and DFS at 1 and 3 years were 84.3% and 63.8% for OS, 55.6% and 30.7% for DFS, respectively. From the analysis of the data, the most significant results indicate that: patients with a lower CEA value <25 ng/mL had an OS of 93.6% and 80.1% at 1 and 3 years, with an average of 36.7 months (CI 95% 33.1–40.3); moreover, patients with a value equal to or greater than 25 ng/mL had a 1-year survival equal to 57.4%, with an average of 13.8 months (CI 95% 9.4–18.2) (*p* < 0.001); adjuvant chemotherapy increases by 3 years the overall survival (OS: 68.6% vs. 49.7%) (*p* = 0.013); localization of the primary tumor affects OS, with a better prognosis for left colon metastases (OS at 42 months: 85.4% vs. 42.2%) (*p* value = 0.056); patients with stage T1 or T2 cancer have a better 3 years OS (92.9–100% vs. 49.7–56.3%) (*p* = 0.696), while the N0 stage results in both higher 3 years OS and DFS than the N + stages (OS: 87.5% vs. 68.5% vs. 24.5%); metachronous metastases have a higher 3 years OS than synchronous ones (80% vs. 47.4%) (*p* = 0.066); parenchymal sparing resections have a better 3 years DFS than anatomical ones (33.7% vs. 0%) (*p* = 0.067); a patient with a parenchymal R1 resection has a much worse prognosis than an R0 (3 years OS: 0% vs. 68.7%) (*p* < 0.001). Conclusions: CEA value of less than 25 ng/mL, localization of the primary tumor in the left colon, primary tumor in stage T1/2 and N0, metachronous presentation, R0 resection, fewer than four metastases, and use of adjuvant chemotherapy are all parameters that in our analysis have shown a correlation with a better prognosis; moreover, the evaluation of the series is in line with the latest evidence in the literature in defining the non-inferiority of minimally invasive and parenchymal sparing treatment compared to the classic laparotomic approach with anatomic resection.

## 1. Introduction

About 25–30% of patients diagnosed with colorectal cancer develop liver metastases (CRLM) during the disease making it the most frequent secondary liver tumor [1,2,3,4]. The incidence of synchronous liver metastasis ranges from 13.8% to 17.1% in epidemiology, while the incidence for metachronous CRLM is reported between 7.6% and 15.1% [5,6,7]. As far as the secondary metachronous metastases, they usually appear early on in the *follow-up* period for the most part: in 76–85.3% they appear in the first year and 83–97.5% within the first 3 years. Furthermore, 30–40% of patients with metachronous CRLM appear to have the disease limited to the liver. About 2% of patients moreover develop liver metastases between 5 to 10 years after the primary tumor resection [8,9].

Historically, metastatic colorectal cancer has been associated with poor survival [10].

Surgical resection has a fundamental role in increasing the chance of survival in these patients [7,11,12]; as a matter of fact, studies have shown that chemotherapy as a monotherapy accounts only for a 5-year OS of 2.2%, while patients receiving *best supportive care* have an even more inauspicious outcome.

Liver resection can achieve 5-year survival rates of above 50%, compared to only around 5% for patients treated with palliative intent [13].

Unluckily, only 7–25% of affected patients have a medical indication for surgery [14,15], also considering intra- and post-operative challenges due to complex surgical resections [16,17,18]. 

However, the outcomes of patients with CRLM have improved significantly over the past few decades due to advances in systemic therapy, locoregional treatment and minimally invasive surgery [12,19,20], each of which have contributed to the expansion of safe hepatic resection [21,22,23,24,25]. 

So, the median five-year survival rate of patients with metastases of colorectal cancer has risen from <10% to 35–40%, and the median overall survival (OS) has increased from <12 months to approximately 42 months [26,27,28].

Some authors reported a 5-year overall survival in patients treated with curative liver resection of 20–60% and 10-year survival of 26–42% [6].

The main factors which influence prognosis are the volume of liver affected by disease, presence of extra-hepatic metastases, low-grade cell differentiation, mesenterial lymph node, CEA levels, male sex and the patient’s age [6,29].

This study’s purpose is to investigate the main prognostic factors correlated with both overall survival (OS) and disease-free survival (DFS), in order to identify patients that could most benefit from liver resection surgery.

## 2. Materials and Methods

The study analyzed 75 patients who underwent surgical treatment for liver metastases secondary to CRC between April 2018 and September 2021 in the general surgery ward at the Ospedal San Jacopo di Pistoia.

All patients were staged with a thoracic-abdomen-pelvis CT; liver volume assessment was carried out in patients who underwent major liver resection. In selected cases liver specific contrast MRI was carried out for diagnostic completion. Intraoperative US was routinely used during hepatic surgery.

Inclusion criteria for this study were having undergone liver resection for synchronous or metachronous liver metastases secondary to colorectal carcinoma with a curative intent.

A careful preoperative assessment of the liver function with prediction of postoperative residual functional liver parenchymal mass was evaluated in patients nominated for major liver resection to minimize the surgical risk. The remnant hepatocyte function was evaluated with an indocyanine green (ICG) retention test [30] and the spatial volumetry of the liver assessed by computed tomography (CT) or magnetic resonance imaging (MRI) enabled the prediction of the future liver remnant volume.

*Follow-up* was carried out with the aid of imaging, especially CT, and oncological markers mainly CEA, every 3–6 months according to patient characteristics and on oncological recommendation [31]. The average *follow-up* lasted 17 months and ranged from 1 to 42 months.

Patient data were collected on an Excel database and retrospectively analyzed using IBM SPSS Statistics analysis software. The patients’ data were obtained in an ethical manner. All of the collected variables, including the baseline characteristics, the perioperative outcomes and the current follow-up were expressed as the mean and standard deviation (M ± SD) or by numbers and percentages.

For the univariate statistical analysis, the overall survival and disease-free survival were calculated by the Kaplan-Meier method. A univariate comparison of survivals was performed by the log-rank test. A *p* value of less than 5% was considered statistically significant. 

The statistical calculations were achieved by the Statistical Package for Social Sciences, ver. 24 (SPSS, Chicago, IL, USA).

## 3. Results

### 3.1. Patient Characteristics

A total of 75 patients met the inclusion criteria for this study. 39 of which were female (52%) and 36 male (48%). The average age was 70 years old, with a range between 33 and 85; by dividing patients into age groups, 63 (84%) were 60 years or older at the time of the surgical procedure, while 12 (16%) were younger than 60 years. Average BMI was 25 kg/m^2^ and ranged between 15.2 and 37.6 kg/m^2^.

Of the 75 patients, only 17 (22.7%) did not have comorbidities, meanwhile for the remaining patients the five most frequent comorbidities in decreasing order were: hypertension (31.1%), diabetes mellitus type II (8.4%), obesity (7.6%), atrial fibrillation (6.7%) and chronic obstructive pulmonary obstruction (4.2%). Furthermore, 24 patients (32%) were ex-smokers, four (5.3%) were current smokers at the time of surgery and 47 (62.7%) denied smoking regularly.

In these patients, the primary tumor site turned out to be a neoplasm of the right colon(including cecum and ascending colon) in 20 patients (26.7%), a primary tumor of the transverse colon in three patients (4%), meanwhile in 26 cases (34.7%) the tumors were located in the left colon (including the descending colon and sigma), in 22 patients (29.3%) the primary tumor was of the rectum, and in just four cases (5.4%) the neoplasm had an unknown origin.

TNM-AJCC 2017 [32] staging of the primary tumor showed that as far as T was concerned one case (1.4%) was T1, 18 cases (25.4%) were T2, 36 cases (50.7%) were T3 and 16 cases (22.5%) were T4; the node involvement showed, 23 patients (32.4%) were N0, 32 (45.1%) were N1 and 16 (22.5%) were N2. In 47 cases (66.2%) grading of the primary tumor was G2 while in 24 cases (33.8%) the staging was G3.

During the study, of the 75 patients included, 39 (52%) resulted as having developed synchronous metastases during the active disease, while 36 (48%) showed the appearance of metastases during follow-up after the treatment of the primary tumor.

Of the patients with synchronous metastases 22 cases (61%) were operated simultaneously for both primary and metastatic resection, in 14 cases (39%) the patients had a delayed surgery; of these five patients (13.9%) had a liver first surgical approach.

Preoperative lab exams showed that average levels of hemoglobin were about 12.4 g/dL (range 7–16.6 g/dL), platelets were 237 10^9^/L (range 22–498 10^9^/L), blood serum creatinine was 0,84 mg/dL (range 0.49–3.18 mg/dL), INR was 1.07 (range 0.89–1.96), and total bilirubin was 0,84 mg/dL (range 0.26–9.8 mg/dL). The average CEA level was 22.7 ng/mL (range 0.3–189 ng/mL) with 45 patients (60%) that had higher levels in respect to the reference values (0–5 ng/mL). Eleven patients (14.7%) also presented with higher levels of CA 19.9 above the reference values (<40 U/mL), with an average of 97 U/mL (range 0.8–1081.2 U/mL).

Upon surgery patients were divided according to their ASA score [33]: three (4%) had a score equal to one, 33 (44%) had a score equal to two, 37 (49.3%) were categorized in the third class and two (2.7%) had a score of four. As far as the type of surgical resection is concerned, 54 patients (72%) had a *parenchymal sparing* resection while the remaining cases were treated with anatomical resections, according to Brisbane’s classification [34], in decreasing order of frequency eight bisegmentectomies (38%), six segmentectomies (28.6%), three right hemihepatectomies (14.3%), two left paramedian sectorectomies (9.4%), one left hemihepatectomy (4.8%) and one extended right hepatectomy (4.8%). According to the volumetric criterion (paragraph 2.11), there were sixteen minor resections and five major resections.

In nine patients (12%) a minimally invasive laparoscopic surgery approach was used with an average duration of 261 min (range 185–429 min); 33% of cases required of a laparotomic conversion. The average length of the laparotomic surgeries was 261 min (range 80–573).

In the laparotomic surgery approach the most common incision in decreasing order were: Makuuchi’s J-shaped incision in 38 cases (55%), a Kocker (right subcostal) incision in 15 cases (21.7%), a midline xiphoid-pubic or xipho-subumbelical in 13 cases (18.9%) and a bi-subscostal incision in three cases (4.3%). The Pringle maneuver was utilized in four cases (5.3%). The prophylactic isolation of the hepatic pedicle was achieved routinely, although the clamping (Pringle’s maneuver) was employed selectively. In the case of important blood loss the intermittent clamping lasting 15 min followed by 5 min of reperfusion was performed. Intraoperative blood transfusions were required in six patients (8%).

At the time of surgery, only one lesion was present in 32 patients (42.7%), fewer than four metastases were removed in 24 cases (32%) and there were four or more metastases in 19 patients (25.3%)

Monolobar metastases were found in 55 patients (73%), 36 (48%) of which were in the right lobe while 19 (25%) affected the left lobe; in the remaining 20 cases (27%) the disease was bilobar.

Radiofrequency ablation was used alongside surgical resection in 20 patients (26.7%) with an average of two lesions ablated (range 1–4) in each surgery in which this technique was used.

Histopathologic examination of the tissue samples gave the following results: 15 patients (20%) showed neoplastic infiltration (R1) of the surgical margin, 10 (66.7%) of which presented with a vascular infiltration (vascular R1) while the remaining 5 (33.3%) had only a parenchymal involvement (parenchymal R1). Furthermore, in R0 patients the distance between the mass and the resection margin was less than 1 mm in 16 (26.7%), 21 patients (35%) presented with a distance between 1–5 mm, meanwhile in 23 cases (38.3%) the distance was equal or greater to 5 mm.

Intensive monitoring in postoperative care was provided for 45 patients (60%). The average for postoperative recovery was on average 9.5 days, with a range of 4–43 days. Oral nutrition was on average started on the third postoperative day (range 2–11 days) and bowel movement usually appeared on the fifth day on average (1–10th day). The Clavien-Dindo index [35] was on average 1.5, with seven patients having noted significant complication (CD III–IV) in the post-OP period. Amongst the most common minor complications the most refered were vomit (35.9%) between the third and eighth PO day, anemia (25.6%), hypotension with fainting (5.1%) and rectorrhagia (5.1%) between the second and third day. Post-OP blood transfusions were necessary in 17 patients (22.7%). Five patients (6.7%) required a second surgery, two due to anastomosis dehiscence, two due to purulent surgical drains and one thoracocentesis due to bilateral pleural effusion. Post-op mortality was three patients (4%).

Current guidelines recommend upfront surgery in patients with initially resectable disease and low operative risk, reserving neoadiuvant chemotherapy for patients with borderline resectable or unresectable disease and high operative risk. Patients undergoing neoadiuvant chemotherapy require close monitoring for tumor response and conversion of CRLM to resectability. The most applied adjuvant chemotherapy protocol involves the administration of 5-fluorouracil (5FU), leucovorin and the addition of oxaliplatin or sometimes irinotecan. Adjuvant chemotherapy was administered in 54 patients (72%) with an average length of 7 months (range 2–22). Neoadjuvant chemotherapy was used in 25 patients (33.3%) with the intent of downsizing. Patient and surgical features are show in Table 1 and Table 2.

### 3.2. Factors Associated with DFS and OS

During follow-up 19 patients (25.3%) died and 32 patients (42.7%) relapsed 20 cases (62.5%) with liver metastases, 8 cases (25%) with lung metastases and peritoneal metastases in the remaining cases. The liver metastases relapses were treated in 26.1% with only chemotherapy and in 17.4% with radiofrequency ablation, in 13% of cases a combined method was used and in 21.7% of cases only a resection was used.

Overall survival of patients at 1 and 3 years was equal to 84.3% and 63.8% respectively with and average of 31.4 months (CI 95% 27.4–35.3); meanwhile disease-free survival at 1 and 3 years was 55.6% and 30.7%, respectively, with a median of 21.9 months (CI 95% 17.5–26.3).

Univariate analyses of factors associated with overall survival and disease-free survival are described in Table 3.

**Figure 1:**
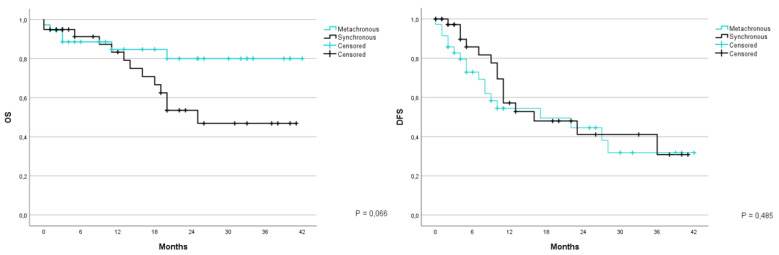
Kaplan-Meier curve for OS and DFS in patients who underwent liver resection for CRLM stratified based on the temporal presentation of metastasis (synchronous/metachronous). OS: overall survival; DFS: disease free survival; CRLM: colorectal liver metastases.

By comparing the survival between patients with synchronous and metachronous metastases it is noticeable is that OS at 1 and 3 years for synchronous metastases is at 83.3% and 46.9%, respectively, with an average survival of 26.9 month (CI 95% 21.4–32.5); while for metachronous metastases the OS at 1 and 3 years is at 84.7% and 80%, with an average survival of 35.2 months (CI 95% 30.2–40.1) (*p* = 0.066). Disease free survival at 1 and 3 years was 97.1% and 30.8% for synchronous metastases and 54.4% and 31.8% for metachronous metastases; with an average of 22.6 months (CI 95% 3.1–16.5) for synchronous metastases and 21.1 months (CI 95% 3.1–14.9) for metachronous metastases (*p* = 0.485) (Figure 1).

**Figure 2:**
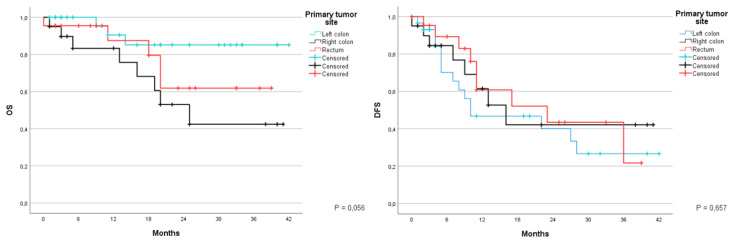
Kaplan-Meier curve for OS and DFS in patients who underwent liver resection for CRLM stratified based on the primary tumor site. (Left colon, right colon, rectum). OS: Overall Survival; DFS: Disease Free Survival; CRLM: Colorectal liver metastases.

Based on the site of the primary tumor it was possible to organize patients into three groups: the left colon, the right colon and the rectum. Analyzing the different global survival at 1 and 3 years of these groups it was noted that an OS of 90.5% and of 85.2% for the left colon, with an average of 37.5 months (95% CI 32.7–42), of 83.3% and of 42.4% for the right colon, with an average of 25.7 (95% CI 18.2–33.2), and of 87.5% and 61% for the rectum with an average of 29.9 (95% CI 23.5–36.4) (*p* value = 0.056) respectively. For DFS the left colon group showed 46.7% and 26.7%, with an average of 19.6 months (CI 95% 12.9–26), the second group showed 61.4% and 42.1% with an average of 22.4 months (CI 95% 13.6–31.3 months), and the third group showed 60.8% and 21.7%, with an average of 22.9 months (CI 95% 15.8–30.1) (*p*-value = 0.657) (Figure 2).

**Figure 3:**
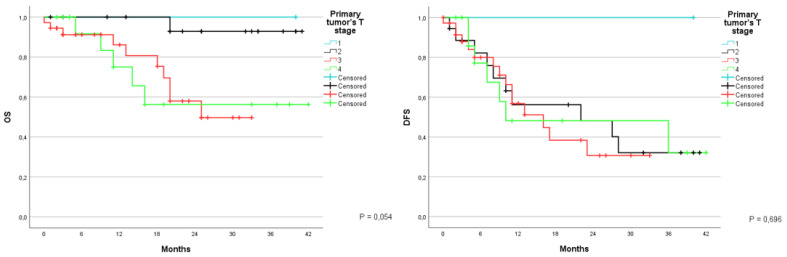
Kaplan-Meier Curve for OS and DFS in patients who underwent liver resection for CRLM stratified according to the primary tumor’s T stage. OS: Overall Survival; DFS: Disease Free Survival; CRLM: Colorectal liver metastasis.

The correlation between survival and tumor T stage for the primary tumor was also analyzed. OS at 1 and 3 years was 100% for patients with T1; 100% and 92.9% respectively for T2; 86.1% at 1 year for T3; 75% and 56.3% at 1 and 3 years for T4 (*p*= 0.054). DFS was 100% respectively at years 1 and 3; 56.2% and 32.1%; 56.9% at year 1; and 48.2% and 32.1 at years 1 and 3 respectively (*p* = 0.696) (Figure 3).

**Figure 4:**
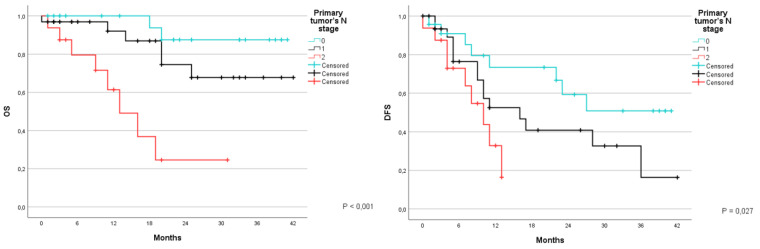
Kaplan-Meier Curve for OS and DFS in patients who underwent liver resection for CRLM stratified according to the primary tumor’s N stage. OS: Overall Survival; DFS: Disease Free Survival; CRLM: Colorectal liver metastasis.

Subsequently the same analyses were carried out with the N parameter. The overall survival at 1 and 3 years was 100% and 87.5% for patients with N0, with an average survival of 38.3 months (CI 95% 34.7–41.8); 92% and 67.7% for patients with N1 with an average survival of 33.9 months (CI 95% 28.4–39.4); 61.4% at 1 year for patients with N2, and an average survival of 15.9 months (CI 95% 0.1–21.9) (*p* < 0.001). The DFS for the same groups was 73.4% and 50.8% respectively, with an average of 28 months (CI 95% 21.1–34.9); 52.5% and 16.3%, with an average of 20.2 months (CI 95% 13.9–26.6); and 32.8% at 1 year, with an average of 8.6 months (CI 95% 6.3–11) (*p* = 0.027) (Figure 4).

**Figure 5:**
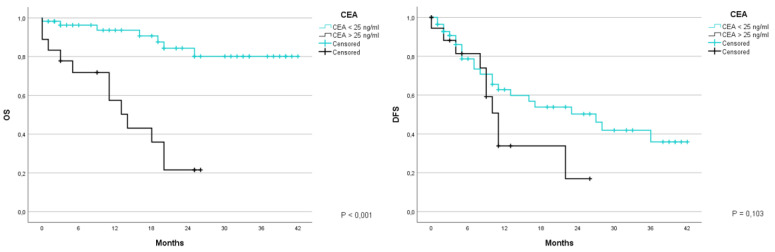
Kaplan-Meier Curve for OS and DFS in patients who underwent liver resection for CRLM stratified according to CEA values. OS: Overall Survival; DFS: Disease Free Survival; CRLM: Colorectal liver metastasis.CEA carcinoembryonic antigen.

Taking into consideration the carcinoembryonic antigen (CEA). various studies were performed to determine the cut-off value that would allow a greater difference in the results. the value chosen was 25ng/mL. The patients with a lower CEA value than the cut-off had an OS of 93.6% and 80.1% at 1 and 3 years, with an average of 36.7 months (CI 95% 33.1–40.3); moreover. patients with a value equal to or greater than 25ng/mL had a 1-year survival equal to 57.4%. with an average of 13.8 months (CI 95% 9.4–18.2) (*p* < 0.001). DFS from disease at 1 and 3 years was 62.8% and 35.9% for the first group with an average of 23.9 moths (CI 95% 18.7–28.9), of 50.7% at 1 year for the second group with an average of 13.1 months (CI 95% 8.6–17.6) (*p* = 0.103) (Figure 5).

**Figure 6:**
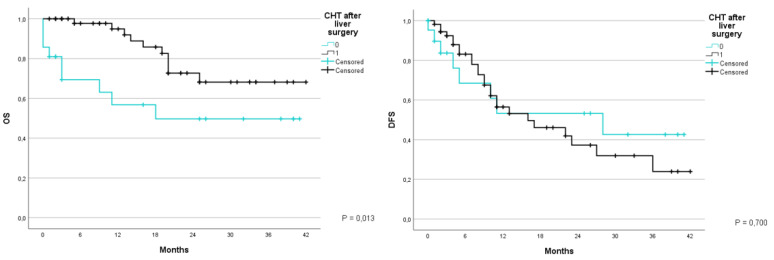
Kaplan-Meier Curve for OS and DFS in patients who underwent liver resection for CRLM stratified according whether the patient had adjuvant chemotherapy or not: Overall Survival; DFS: Disease Free Survival; CRLM: Colorectal liver metastasis; CHT: chemotherapy.

Further studies have been carried out to analyze the correlation between survival and adjuvant chemotherapy. We subdivided the patients in two groups, the first group which did not undergo treatment (0) and the second which did (1). The global survival at 1 and 3 years in the 1st group was 56.8% and 49.7% respectively, with an average of 23.3 months (CI 95% 14.8–31.8), compared to 94.9% and 68.2% respectively for the second group. with an average of 34.1 months (CI 95% 29.9–38.2) (*p* = 0.013). During the same period the DFS was 53.2% and 42.6% for those who did not undergo treatment, with an average of 22.9 months (CI 95% 13.9–31.9), and of 56.5% and 23.9% for those who underwent chemotherapy with an average of 20.9 months (CI 95% 15.9–25.9) (*p* = 0.700) (Figure 6).

**Figure 7:**
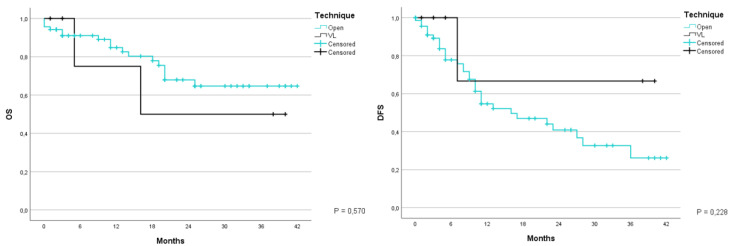
Kaplan-Meier Curve for OS and DFS in patients who underwent liver resection for CRLM stratified according to type for surgical technique used. OS: Overall Survival; DFS: Disease Free Survival; CRLM: Colorectal liver metastasis. Open; VL-video laparoscopy.

Comparing the type of surgery technique chosen, the patients who were operated with a laparotomic hepatic resection had a global survival at 1 and 3 years of 84.8% and 64.7% respectively with an average of 31.7 months (CI 95% 27.7–35.8months); while those who underwent a laparoscopic procedure had an OS of 75% and 50%, with an average of 25.3 months (CI 95% 10.3–40.2) (*p* = 0.570). Moreover, DFS in the same period was 54.7% and 26.2% for the first group with an average of 21 months (CI 95% 16.5–25.5), and 66.7% for the second group, with an average of 29 months (CI 95% 11.4–46.6) (*p* = 0.228) (Figure 7).

**Figure 8:**
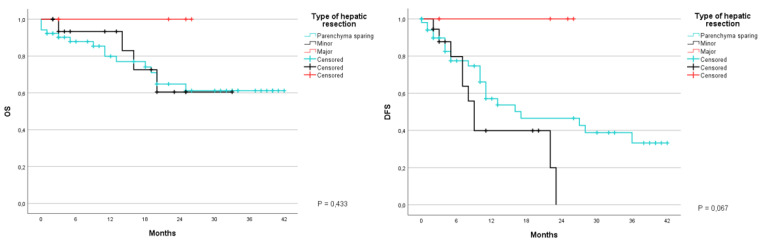
Kaplan-Meier Curve for OS and DFS in patients who underwent liver resection for CRLM stratified according to type of hepatic resection. OS: Overall Survival; DFS: Disease Free Survival; CRLM: Colorectal liver metastasis.

The patients who participated in this study underwent various types of hepatic resections grouped in three categories: parenchymal sparing (atypical resection), minor anatomical resection (sub-segmentectomy, segmentectomy, or bi-segmentectomy), and major resections (removal of more than three segments). Global survival was 79.9% and 61.2% at 1 and 3 years for atypical resections, 93.3% at 1 year for minor resections and 100% at 1 year for major resections (*p* = 0.433). DFS however turned out to be 57.1% and 33.2% at 1 and 3 years for the first group, 39.9% and 0% for the second group in the same time period and 100% at 1 year for the third group (*p* = 0.067) (Figure 8).

**Figure 9:**
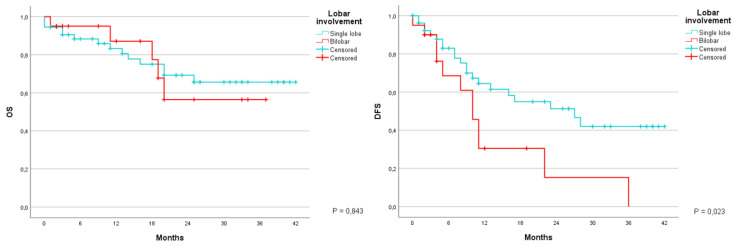
Kaplan-Meier Curve for OS and DFS in patients who underwent liver resection for CRLM stratified according to lobar involvement. OS: Overall Survival; DFS: Disease Free Survival; CRLM: Colorectal liver metastasis.

By analyzing the survival in patients according to which hepatic lobe contained the liver metastases the following results were noted. OS at 1 and 3 years in metastases confined to a single lobe was 83.3% and 65.6%, with an average of 31.5 months (CI 95% 26.9–36.1); patients with bilobar metastases were 87.1% and 56.4%, with an average 27.6 months (CI 95% 21.2–34) (*p* = 0.843). The DFS at the same time was 64.5% and 42%, with an average of 24.6 months (CI 95% 19.4–29.8) for the first group, and 30.5% and 0%, with an average of 13.7 months (CI 95% 7.1–20.2) for the second group (*p* = 0.023) (Figure 9).

**Figure 10:**
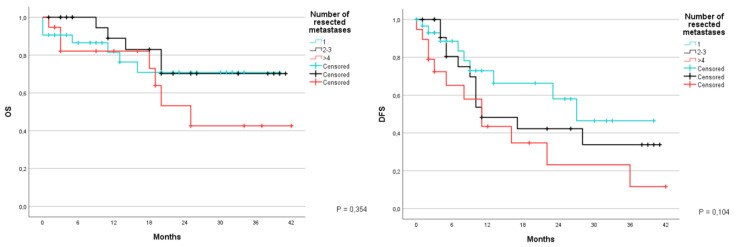
Kaplan-Meier Curve for OS and DFS in patients who underwent liver resection for CRLM stratified according to number of lesions removed. OS: Overall Survival; DFS: Disease Free Survival; CRLM: Colorectal liver metastasis.

Another parameter that we analyzed was the number of resected metastases during surgery. Patients were divided into three groups: the first group included patients with the resection of only one metastasis; the second group included those that had two or three lesions removed; and the third group of patients had four or more metastases resected. Overall survival at 1 and 3 years was equal to 86.5% and 70.9% for the first group, with an average of duration of 30.6 months (CI 95%24.7–36.6). 88.9% and 70.2% for the second group, with an average of 33.3 months (CI 95% 27.5–39). 82.1% and 42.6% for the third group, with an average of 26.5 months (CI 95%18.3–34.6) (*p* = 0.354). As far as the DFS is concerned the data collected were the following: 72.9% and 46.4%, with an average of 25.9 months (CI 95% 19.1–32.8); 48.3% and 33.8%, with an average of 21.2 months (CI 95% 14.1–28.2); 43.4% and 11.6%, with an average of 15.9 months (CI 95% 8.4–23.5) (*p* = 0.104) (Figure 10).

**Figure 11:**
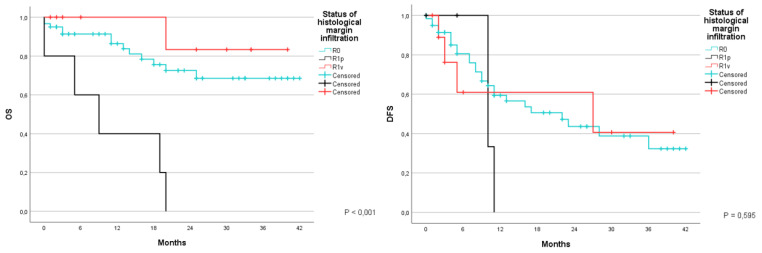
Kaplan-Meier Curve for OS and DFS in patients who underwent liver resection for CRLM stratified according to the status of histological margin infiltration. OS: Overall Survival; DFS: Disease Free Survival; CRLM: Colorectal liver metastasis; R1p: parenchymal R1; R1v: vascular R1.

Another aspect that was studied was whether or not the surgical margins showed signs of infiltration during the histopathogical examination. Patients were divided yet again in three groups: the first group R0 included patients in which the lesion was completely removed; the second group included patients with a vascular R1; and the third group included patients with parenchymal R1, that is to say when an altered cell morphology was found in the liver tissue at a microscopical level. OS at 1 and 3 years was 86.4% and 68.5%, with an average of 32.7 months (CI 95% 28.4–37); the second group was 100% and 83.3%, with an average of 36.7 months (CI 95% 30.7–42.6); meanwhile the third group had an OS at 40% and 0%, with an average of 10.6 months (CI 95% 2.9–18.3) (*p* < 0.001). DFS during the same time was 59.4% and 32.3% with an average of 22.6 months (CI 95% 17.7–27.6) in patients with R0; 61% and 40.6% with an average of 23.1 months (CI 95% 11–35.2) for patients with R1v; and 0% with an average of 10.3 months (9.7–10.9) in patients with R1p (*p* = 0.595) (Figure 11).

**Figure 12:**
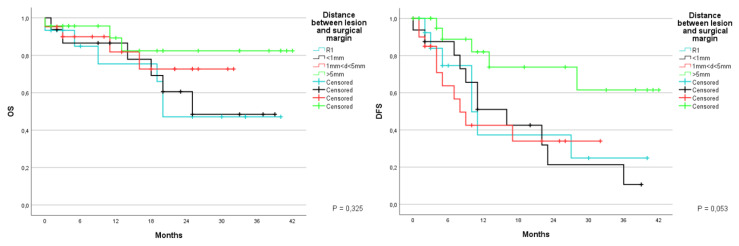
Kaplan-Meier Curve for OS and DFS in patients who underwent liver resection for CRLM stratified according to distance between lesion and surgical margin. OS: Overall Survival; DFS: Disease Free Survival; CRLM: Colorectal liver metastasis.

Survival was also studied according to the histological distance between the neoplasm and resection margin. Patients were divided into four groups: the first group included those in which the neoplasm was seen on the margin. meaning R1; the second group included patients with a distance inferior to 1 mm; the third group included those with a distance between 1–5 mm; and the fourth group included those where the margin was greater than 5 mm. OS was respectively at 1 and 3 years of 74.4% and 47.1% for the first group, with an average survival of 25.7 months (CI 95% 17.3–34.1); 86.5% and 48.5% at 1 and 3 years for the second group, with an average of 26.7 months (CI 95% 19.3–34.2); 81.8% at 1 year for the third group, with an average of 25.8 months (CI 95% 20.4–31.1); 89.3% and 82.4% at 1 and 3 years for the fourth group, with an average of 36.2 months (CI 95% 30.2–42.2) (*p* = 0.325). The disease free survival for the same period was 37.3% and 24.9% in the first group, with an average of 18 months (CI 95% 8.7–27.4); 51% and 10.6% for the second group, with an average of 17.6 months (10.9–24.3); 42.5% at 1 year in the third group, with an average of 15.1 months (CI 95% 8.7–21.6); 82% and 61.5% in the fourth group, with an average of 31.5 months (CI 95% 17.5–26.3) (*p* = 0.053) (Figure 12).

## 4. Discussion

Five-year survival in patients with CRC with liver metastases is 25%, meanwhile in CRC without liver metastases, the 5-year survival is 75%, this underlines the fact that liver metastases represent a main factor in conditioning patient prognosis [36,37]. Surgical treatment is still the best option, liver resection allows 5-year OS to reach about 50%, which is ten times greater than patients treated with palliative treatment [6].

Surgical resection of CRLM remains the gold standard and the best opportunity for long-term survival [38,39]. For a select cohort of patients with CRLM, surgical resection can be curative. Refinements in the understanding of surgical anatomy along with surgical technique have resulted in an expanded assortment of available surgical approaches and have expanded what is considered resectable [40].

It is therefore of fundamental importance to identify and analyze those factors that affect survival in patients that undergo hepatic resection, in order to select and choose the best treatment option for each case. It is important to discuss each single case at a multidisciplinary group, as to be able to better personalize each treatment option, increasing long-term survival as reported in the literature [7,41,42,43].

Taking into consideration that the only current curative treatment option remains hepatic resection, guidelines have been modified and expanded in time in order to increase the number of patients that can benefit from this treatment [44]. At the moment the importance no longer lies with the number, size and diffusion of the metastases, as seen in our study of 19 patients with three or more metastases and 20 cases in which the disease affected both lobes, but rather the only crucial criterion is the possibility to perform a complete resection while preserving sufficient future remanent liver (FRL) [45,46,47]. As a matter of fact, according to the literature [48], and also from our studies, the surgical margin resection is that which has the most influence on patient prognosis with an OS of 42 months equal to 68.7% in patients who had undergone resection R0, while no patient with an R1p had a survival of more than 20 months after surgery. Another interesting fact is the difference in OS and DFS between R0 and R1v; it is therefore recommended that in the case of vascular infiltration, the liver resection be carried out with a potential vascular structure reconstruction if necessary.

If a complete R0 resection is not possible as a first approach, or FRL is insufficient, it is useful to consider treatment options that increase the possibility of surgical resection such as PVE, two-stage hepatectomy, or ALPPS [49,50,51,52,53]. Even neoadjuvant chemotherapy has been shown to be useful in-patient preparation for surgical resection [54,55], nonetheless, when considered as a prognostic factor it had scarce impact on survival [56,57], as a matter of fact our study showed that OS at 40 months was similar between patients who underwent neoadjuvant treatment and those who didn’t (65.1% vs. 63.1%), and likewise for the DFS (23% vs. 37.7%).

There are various systems that analyze CRC patient prognosis such the *Fong score* and the *GAME score* [58,59]. The GAME score was calculated by allocating points to each patient according to the presence of these predictive factors: KRAS-mutated tumors (1 point); carcinoembryonic antigen level 20 ng/mL or more (1 point), primary tumor lymph node metastasis (1 point); a tumor burden score between 3 and 8 (1 point) or 9 and over (2 points); and extrahepatic disease (2 points). Patients were assigned to low-risk (GAME: 0–1), medium-risk (GAME: 2–3) and high-risk (GAME: ≥ 4) categories. The 5-year OS was 11% for the high-risk group (GAME ≥ 4), while it was 73% for the low-risk group (score 0–1). The study performed at Johns Hopkins showed the GAME score as superior to the Fong score.

In our study we investigated those factors that had a greater influence on long-term survival in patients who had surgical treatment for CRLM. Three categories have been determined: those regarding the patients, those regarding the neoplastic disease and those regarding the surgical procedure.

As far as the patient characteristics are involved, the impact of comorbidities, CEA values and whether patients had post-OP adjuvant chemotherapy were looked at with greater attention. Patients with pathologies other than the neoplastic disease had a worse outcome with an OS at 40 months of 60.1% compared to 73.7% in patients without comorbidities due to a worse performance status and major post-OP complications, this data however has not been proven to be statistically significant. Another factor that has turned out to be statistically significant (*p* < 0.001) for both OS and DFS is the CEA levels; patients with CEA levels greater than 25ng/mL had a survival of 26 months, which is inferior compared to those patients with CEA levels below this value (21.5% vs. 80.2%), and also a greater risk of relapse (55.6% vs. 40.4%). As far as adjuvant chemotherapy, according to the current studies, there is no absolute indication about the use of this treatment option in patients who have had surgical liver resection [60], however, in our data, patients who had post-OP chemotherapy had a greater OS (68.6% vs. 49.7%), with statistically significant results (*p* = 0.013), as was similarly seen in Bartolini et al. [61]. Instead, the results related to the DFS were not statistically significant and showed a greater disease-free survival in those patients who did not undergo post-OP chemotherapy (43.1% vs. 24.2%), it is important to note that these values could be subject to *bias* due to the fact the most patients recommended for chemotherapy were those with a higher chance of relapse.

With regards to the factors of the disease itself, we can identify two main groups, i.e., the characteristics of the primary tumor and the characteristics of the metastases. For the primary tumor, we analyzed the site of the neoplasm, the TNM stage, and grading; while for the metastases, we analyzed the temporal presentation, number, and lobe involvement. The site of the primary tumor was an important prognostic factor, as was already seen in the literature [6], and it was confirmed in our study in which OS in patients with left-side colon cancer at 42 months was 85.4% compared to 42.4% of patients with right colon cancer. The results were on the threshold of statistical significance (*p* = 0.056). It is probably due to the fact that metastases due to right-side colon cancer are diagnosed late, when the disease is already widespread, and they have a molecular profile of the tumor. Even the study of the primary tumor has an important impact on the prognosis of surgically resected patients, in our study we were able to sort patients in to two groups: the first was composed of patients with T1 or T2 stage neoplasm that have OS greater than 90%, while the second group is composed of patients with T3 and T4, with an OS inferior to 60%. This study was on the edge of statistical significance (*p* = 0.054), as opposed to the DFS that showed a similar relapse risk, with a DFS below 35% for all stages except T1. Other statistically significant results (*p* < 0.05) emerged from the analyses of the N parameter; a correlation between OS and DFS was seen, with minor stages showing a greater survival compared with N+ (87.5% vs. 68.5% vs. 24.5%); which can be tied to both a greater weight of disease and a greater probability of relapse. Grading was also seen to be correlated with prognosis, patients with G2 had a greater OS than patients with G3 (76.6% vs. 43.8%), even though neoplasms with a greater differentiation showed less DFS (22.6% vs. 49.1%). As pertains to the metastatic characteristics, the results are similar with what is already present in the literature [27], what emerged in our study was that synchronous metastases had a worse OS compared to the metachronous metastases (47.4% vs. 80%), even if there are no differences in the DFS, this shows that surgical resection is also to be recommended, even when metastases appear sometime after the resection of the primary tumor. The number of metastases however, showed no difference amongst patients with fewer than three metastases (OS 70%) while those with four or more metastases showed a significantly worse outcome (43.8%), and a greater risk of relapse (37.5% vs. 63.1%). Finally, as far as which hepatic lobe was involved, our study did not demonstrate a statistically significant difference in the results, with an OS greater than 50% for all of the groups; the DFS however, showed a greater value in patients with bilobar metastases, 0% at 36 months compared to 42% in patients whose metastases involved only one lobe, these values were statistically significant (*p* = 0.02).

The assessment of the surgical procedure characteristics has permitted the analysis of three factors: the type of surgical approach, the type of procedure, and the distance between surgical margins and the disease. As far as it pertains to the surgical approach it is important to highlight that laparoscopic technique is being used more every day in the treatment of CRLM [38]. Our study only included six patients treated with the VL approach; therefore results are not very significant, even though they show a non-inferiority of this approach compared to laparotomy, as far as the DFS in concerned (66.7% vs. 26.4%) and an OS nonetheless above 50%. Based on the different types of procedures two groups were compared, those with an anatomical resection and those with parenchymal sparing. The results are similar with what has already been seen in the literature [62,63], which show a non-inferiority of non-anatomical resection compared to the classic approach, with an OS greater than 60% for both methods and a DFS equal to 33.7% for wedge resections compared to the 0% at 24 months with anatomical resections. It is important to note that as opposed to other studies, our patients treated with major liver resections had an OS and DFS of 100% in 24 months; this could be a result of the small number of patients treated with this technique. A further indication in using the non-anatomical technique is given by the data correlating the prognosis and distance between disease and surgical margins. According to what is seen in various studies [64], this factor influences the OS when the disease is less than 1 mm away from the surgical margin, while when the distance is greater the overall survival is greater, values go from an OS below 50% to above 70%. Nevertheless, it is advisable to try and keep a margin of 5mm during surgery, especially to decrease the risk of relapse (21.7% vs. 56.8%).

Open and minimally invasive hepatobiliary surgery requires a well-trained surgeon and specific competences [65]. Nevertheless, minor liver resections could be performed by general surgeons. Therefore, we would like to underline that minor and major resections either in open and minimally invasive surgery need a different learning curve, according to literature [65]. Indeed, operative time and blood loss, are often used as learning curve predictive factors, but they are not matched to the patients’ characteristics, post operative complications, and tumor locations. In addition, our experience was based on more than one surgeon with different established techniques in distinct surgical fields.

We compared our findings to the relevant literature: we reported a longer length of stay, postoperative transfusion, and complications rate, with the superimposable R0 rate when compared to medium and high-volume center results [6,23,66,67,68,69]. Similar data were reported according to the OS and DFS [6,23,66,67,68,69].

### Limitations

This study has its limits. First of all, the retrospective nature influences the significance of the data, because the patient analysis is subject to selection bias and unidentified or unknown features; furthermore, the monocentric view of this study conditions a relatively small group of patients, limiting the general final results; lastly the limited duration of *follow-up* does not allow to evaluate important results, such as 5- and 10-year survival, important endpoints especially for the risk of relapse in time. It would be auspicial to increase the number of patients selected and indicated for surgery and increasing the follow-up period to reach a greater statistical significance.

## 5. Conclusions

Management of patients with hepatic metastases secondary to colorectal cancer is remarkably complex due to the heterogeneity of the patients, the diversity in the treatments and the constant updates in treatment in the field. It is evident that a multidisciplinary evaluation and personalized treatment is necessary for the ideal management of the single patients. Numerous factors have been proposed as prognostic elements correlated with the global survival and DFS, some scores (Fong and GAME) are used to stratify prognosis in these patients. The absence of comorbidity, CEA values below 25 ng/mL, left colon primary tumor, primary tumor with TNM stage T1/2 and N0, the presence of metachronous hepatic metastases, vascular resection R0 or R1, and adjuvant chemotherapy are all parameters that showed a correlation with a better prognosis in our study; and vice versa, we would also like to highlight that the number of metastases that do not have an effect on prognosis. The results that emerged from our study are comparable with the latest studies that define the non-inferiority of mini-invasive treatments and parenchymal sparing compared to the traditional laparotomic approach with anatomical resection. It is important to emphasize that none of the factors analyzed was associated with such an unfavorable prognosis to advise against surgical treatment. Despite the important progress, it is still unclear what could be the best strategy in the management of patients affected by CRLM with an unfavorable prognosis, further clinical research on the population is necessary.

## Figures and Tables

**Table 1:** Characteristics of the patients.

Patient’s Features	*n*
Male	36
Female	39
Timing of metastases presentation/treatment	
Synchronous	39
Metachoronous	36
Comorbidities	
No	17
Yes	58
Site of primary tumor	
Right colon	20
Left colon	29
Rectum	22
T stage	
1	1
2	18
3	36
4	16
N stage	
0	23
1	32
2	16
Grading	
2	47
3	24
CEA value	
<25 ng/mL	57
≥25 ng/mL	18
CHT before liver surgery	
No	50
Yes	25
CHT after liver surgery	
No	21
Yes	54

**Table 2:** Surgical Features.

Surgical Features	*n*
Surgery technique	
Open	69
Laparoscopy	6
Type of surgery	
Parenchyma sparing	54
Minor	16
Major	5
RFA combined surgery	
No	55
Yes	20
Hepatic lobe involved	
Single	55
Bilobar	20
Resected lesions	
1	32
2–3	24
≥4	19
Margin status	
R0	60
R1v	10
R1p	5
Distance between lesion and margin of resection	
R1	15
<1 mm	16
1 mm < d < 5 mm	21
≥5 mm	23

**Table 3:** Univariate analyses of the factors associated with overall survival and disease-free survival.

		OS				DFS			
	*n*	1 y (%)	3 y (%)	Median (Months, CI)	*p* Value	1 y (%)	3 y (%)	Median (Months, CI)	*p* Value
Timing of metastases presentation/treatment					0.066				0.485
Synchronous	39	83.3	46.9	26.9 (21.4–32.5)		97.1	30.8	22,6 (3.1–16.5)	
Metachronous	36	84.7	80.0	35.2 (30.2–40.1)		54.4	31.8	21.1 (3.1–14.9)	
Comorbidities					0.636				0.686
No	17	81.9	73.7	31.4 (23.9–38.8)		60.8	27.0	21.6 (13.3–29.9)	
Yes	58	84.5	59.8	30.7 (26.1–35.3)		53.6	34.5	21.9 (16.8–27.1)	
Site of primary tumor					0.056				0.657
Right colon	20	83.3	42.4	25.7 (18.2–33.2)		61.4	42.1	22.4 (13.6–31.3)	
Left colon	29	90.5	85.2	37.5 (32.7–42.2)		46.7	26.7	19.6 (12.9–26.3)	
Rectum	22	87.5	61.9	29.9 (23.5–36.4)		60.8	21.7	22.9 (15.8–30.1)	
T stage					0.054				0.696
1	1	100	100	NE		100	100	NE	
2	18	100	92.9	NE		56.2	32.1	NE	
3	36	86.1	NE	NE		56.9	NE	NE	
4	16	75.0	56.3	NE		48.2	32.1	NE	
N stage					<0.001				0.027
0	23	100	87.5	38.3 (34.7–41.8)		73.4	50.8	28 (21.1–34.9)	
1	32	92.0	67.7	33.9 (28.4–39.4)		52.5	16.3	20.2 (13.9–26.6)	
2	16	61.4	NE	15.9 (10.1–21.9)		32.8	NE	8.6 (6.3–11)	
Grading					0.136				0.148
2	47	83.5	76.5	33.7 (29.3–38.2)		51.4	22.4	19.8 (14.7–24.9)	
3	24	95.8	42.6	28.1 (21.4–34.8)		64.4	49.1	25.9 (17.9–33.9)	
CEA value					<0.001				0.103
<25 ng/mL	57	93.6	80.1	36.7 (33.1–40.3)		62.8	35.9	23.9 (18.7–28.9)	
≥25 ng/mL	18	57.4	NE	13.8 (9.4–18.2)		50.7	NE	13.1 (8.6–17.6)	
CHT before liver surgery					0.936				0.278
No	50	86.9	62.6	31.4 (26.3–36.6)		59.9	37.3	23.9 (18.1–29.8)	
Yes	25	80.3	65.1	30.6 (24.6–36.6)		49.9	22.8	19.1 (12.6–25.5)	
CHT after liver surgery					0.013				0.700
No	21	56.8	49.7	23.3 (14.8–31.8)		53.2	42.6	22.9 (13.9–31.9)	
Yes	54	94.9	68.2	34.1 (29.9–38.2)		56.5	23.9	20.9 (15.9–25.9)	
Surgery technique					0.570				0.228
Open and converted	69	84.8	64.7	31.7 (27.7–35.8)		54.7	26.2	21 (16.5–25.5)	
Laparoscopy	6	75.0	50.0	25.3 (10.3–40.2)		66.7	66.7	29 (11.4–46.6)	
Type of surgery					0.433				0.067
Parenchyma sparing	54	79.9	61.2	NE		57.1	33.2	NE	
Minor	16	93.3	NE	NE		39.9	0	NE	
Major	5	100	NE	NE		100	NE	NE	
RFA combined surgery					0.961				0.25
No	55	81.4	64.9	30.6 (25.9–35.3)		60.6	39.0	23.8 (18.3–29.4)	
Yes	20	89.5	58.4	30.8 (23.7–37.9)		47.8	16.4	18.1 (11.8–24.4)	
Hepatic lobe involved					0.843				0.023
Single	55	83.3	65.6	31.5 (26.9–36.1)		64.5	42.0	24.6 (19.4–29.8)	
Bilobar	20	87.1	56.4	27.6 (21.2–34.1)		30.5	0	13.7 (7.1–20.2)	
Resected lesions					0.354				0.104
1	32	86.5	70.9	30.6 (24.7–36.6)		72.9	46.4	25.9 (19.1–32.8)	
2–3	24	88.9	70.2	33.3 (27.5–39)		48.3	33.8	21.2 (14.1–28.2)	
≥4	19	82.1	42.6	26.5 (18.3–34.6)		43.4	11.6	15.9 (8.4–23.5)	
Margin status					<0.001				0.595
R0	60	86.4	68.5	32.7 (28.4–37)		59.4	32.3	22.6 (17.7–27.6)	
R1v	10	100	83.3	36.7 (30.7–42.6)		61.0	40.6	23.1 (11–35.2)	
R1p	5	40.0	0	10.6 (2.9–18.3)		0	0	10.3 (9.7–10.9)	
Distance between lesion and margin of resection					0.325				0.053
R1	15	75.4	47.1	25.7 (17.3–34.1)		37.3	24.9	18 (8.7–27.4)	
<1 mm	16	86.5	48.5	26.7 (19.3–34.2)		51.0	10.6	17.6 (10.9–24.3)	
1 mm < d < 5 mm	21	81.8	NE	25.8 (20.4–31.1)		42.5	NE	15.1 (8.7–21.6)	
≥5 mm	23	89.3	82.4	36.2 (30.2–42.2)		82.0	61.5	31.5 (17.5–26.3)	

NE = not evaluable; TNM stage according to the AJCC 7th edition; CHT = chemotherapy; minor/major = minor/major hepatectomies; RFA = radiofrequency ablation.

## Data Availability

The data used to support the findings of this study are available from the corresponding author upon request.
